# Schistosomiasis in Zambia: a systematic review of past and present experiences

**DOI:** 10.1186/s40249-018-0424-5

**Published:** 2018-04-30

**Authors:** Chester Kalinda, Moses J. Chimbari, Samson Mukaratirwa

**Affiliations:** 1 School of Nursing and Public Health, College of Health Sciences, Howard College Campus, University of KwaZulu-Natal, Durban, South Africa; 2 College of Health Sciences, Howard College Campus, University of KwaZulu-Natal, Durban, South Africa; 3 School of Life Sciences, College of Agriculture, Engineering and Science, Westville Campus, University of KwaZulu-Natal, Durban, South Africa

**Keywords:** *Biomphalaria*, *Bulinus*, Epidemiology, Ecology, Literature review, Schistosomiasis, *Schistosoma haematobium*, *Schistosoma mansoni*, Zambia

## Abstract

**Background:**

The speedy rate of change in the environmental and socio-economics factors may increase the incidence, prevalence and risk of schistosomiasis infections in Zambia. However, available information does not provide a comprehensive understanding of the biogeography and distribution of the disease, ecology and population dynamics of intermediate host snails. The current study used an information-theoretical approach to understand the biogeography and prevalence schistosomiasis and identified knowledge gaps that would be useful to improve policy towards surveillance and eradication of intermediate hosts snails in Zambia.

**Methods:**

To summarise the existing knowledge and build on past and present experiences of schistosomiasis epidemiology for effective disease control in Zambia, a systematic search of literature for the period 2000–2017 was done on PubMed, Google Scholar and EBSCOhost. Using the key words: ‘Schistosomiasis’, ‘*Biomphalaria*’, ‘*Bulinus*’, ‘*Schistosoma mansoni*’, ‘*Schistosoma haematobium*’, and ‘Zambia’, in combination with Booleans terms ‘AND’ and ‘OR’, published reports/papers were obtained and reviewed independently for inclusion.

**Results:**

Thirteen papers published in English that fulfilled the inclusion criteria were selected for the final review. The papers suggest that the risk of infection has increased over the years and this has been attributed to environmental, socio-economic and demographic factors. Furthermore, schistosomiasis is endemic in many parts of the country with infection due to *Schistosoma haematobium* being more prevalent than that due to *S. mansoni*. This review also found that *S. haematobium* was linked to genital lesions, thus increasing risks of contracting other diseases such as HIV and cervical cancer.

**Conclusions:**

For both *S. haematobium* and *S. mansoni*, environmental, socio-economic, and demographic factors were influential in the transmission and prevalence of the disease and highlight the need for detailed knowledge on ecological modelling and mapping the distribution of the disease and intermediate host snails for effective implementation of control strategies.

**Electronic supplementary material:**

The online version of this article (10.1186/s40249-018-0424-5) contains supplementary material, which is available to authorized users.

## Multilingual abstract

Please see Additional file 1 for the translation of the abstract into the five official working languages of the United Nations.

## Background

Increasing evidence suggests that temperature rise may increase the spread of schistosomiasis [1, 2]. Nevertheless, the net effects of environmental changes involving temperature on the ecology of intermediate host (IH) snails remain a challenge to evaluate. Various laboratory experiments and statistical models continue to examine the potential effect of environmental changes on schistosomiasis [3–7]. These studies have provided important information on risk factors that influence *Schistosoma* infection even at micro-geographical scales [8]. In contrast, the prevalence of infection continues to be high, especially among poor and marginalised communities [9], thus indicating the need for increased health education, and the monitoring and surveillance of IH snail population dynamics.

Zambia is a country found in Southern Africa. It is landlocked and has 10 provinces. The burden of schistosomiasis in the country is still high despite efforts to reduce it [10, 11]. Schistosomiasis, commonly known as bilharzia, is caused either by *Schistosoma haematobium* or *S. mansoni*, and has been reported from various localities since the pre-independence era of the development of the healthcare system [12–16]. Several studies [17, 18] have reported cases of schistosomiasis in various parts of the country. Furthermore, earlier studies [15, 19] conducted in Siavonga, a town along the shores of Lake Kariba in the southern part of the country, observed an increase in the prevalence of *S. mansoni* and *S. haematobium* between 1993 and 1998. In the middle 1980s, certain parts of southern province had a prevalence rate of 57.9% [12].

Zambia has been experiencing changes in its environmental and socio-economic status [20]. These changes coupled with the predicted effect of climate change on IH snails [2–6, 21, 22] may alter the risk of *Schistosoma* infection [23, 24]. Currently, disease control strategies depend on mass drug administration (MDA) strategies [25, 26]. However, cases of re-infection and drug efficacy continue to be debated [27, 28] and this may be exacerbated by future climate change which may potentially proliferate *Schistosoma* production [1, 2, 7].

Taken together, these issues suggest the need to improve existing knowledge on schistosomiasis and identify knowledge gaps on the epidemiology of the disease. To achieve this, a systematic review and synthesis of literature was conducted to improve our understanding of the epidemiology and biogeography of schistosomiasis in Zambia. Existing evidence was summarised and research gaps for the control of schistosomiasis and IH snails were identified.

## Methods

### Search criteria

**Fig. 1:**
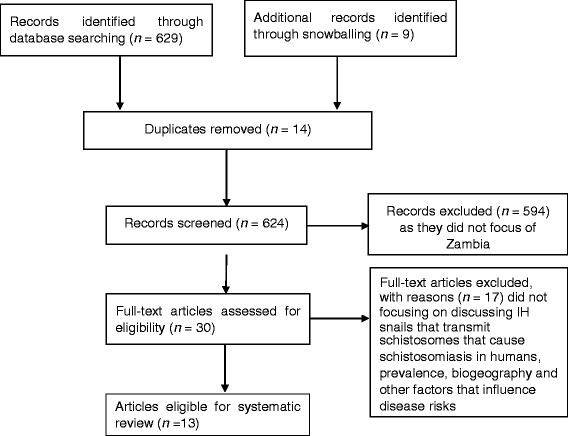
Selection criteria for articles used in the review

We carried out a systematic literature search on PubMed, Google Scholar and EBSCOhost for the period of 2000–2017 (see Fig. 1). The search considered studies that focused on human schistosomiasis and IH snails, *Bulinus* and *Biomphalaria* spp. and parasites, *S. haematobium* and *S. mansoni* in Zambia. The search was based on the combination of the following terms and Boolean operations: schistosomiasis AND *Bulinus* OR *Biomphalaria* snails AND *Schistosoma haematobium* OR *Schistosoma mansoni* in Zambia. More literature was also obtained using the snowballing technique: bibliographies or reference lists of previous reviews or similar studies that had been published in English were examined. Literature on studies that were conducted in other countries such as Zimbabwe but with data collected in Zambia were also obtained and used to strengthen the discussion and understanding of the bionomics of snails and the epidemiology of schistosomiasis. The search excluded articles that reported on schistosomes that infect ruminant animals. Articles that reported on *S. mattheei*, *S. leiperi* and *S. margrebowiei* were also excluded.

The search criteria showed that most of the information on the bionomics of IH snails was from studies conducted in the 1950–1980 period [29]. This is an indication of a paucity of information on the epidemiology of the disease, which includes ecological changes that may have happened because of increased anthropological activities. Therefore, the search looked for articles that discussed disease risks in relation to IH snail bionomics, human-related, and environmental and climatic factors (see Fig. 1).

## Results

### Search results

The search procedure and results obtained are shown in Fig. 1. The search got 629 hits, with an additional nine articles added from snowballing to make a total of 638 articles. However, 625 articles were considered ineligible as they did not meet the inclusion criteria. The relevant articles were categorised based on their study focus. The categories were: malacology, socio-economics/demographics, clinical-based and modelling. The main findings from these articles are summarised in Table 1.

**Table 1:** Characteristics, objectives and outcomes of schistosomiasis studies focusing on Zambia that have been included in the review

Author and Year	Study objectives	Type of study	Study focus	Study location	Major outcomes of study
Mubila & Rollinson (2002) [30]	The study explored the prevalence of urinary schistosomiasis in school children from three disparate areas of Zambia. Furthermore, the compatibility of snails with schistosome parasites from different areas was also investigated.	Cross section and experimental	Parasitological and snail biology	Lake Kariba, Lake Bangweulu and Lusaka	1. *Schistosoma haematobium* infection prevalence was 0% around Lake Bangweulu and 76% around Lake Kariba. 2. F1 progeny snails were highly compatible with the parasite from the local area. The snails were also compatible with all strains of *S. haematobium* collected and tested from different areas. 3. Lake Bangweulu was observed to be an area of low endemicity however, the snails from this area were compatible with straits of *S. haematobium* from both Bangweulu and other areas.
Chimbari et al. (2003) [41]	To explore the differences in the prevalence and intensities of *Schistosoma haematobium* and *S. mansoni* transmission in Lake Kariba and Siavonga	longitudinal study	Parasitological and snail ecology	Siavonga	1. The prevalence of *S. haematobium* and *S. mansoni* infection among school children in Siavonga was 19.4% and 33.5%, respectively. 2. This was higher than that observed in Lake Kariba separated by a distance of 10 km. 3. Better water and sanitation facilities were observed to be the major factor for reduced prevalence of schistosomiasis in Kariba compared to Siavonga.
Simoonga et al. (2008) [37]	The study investigated the risk factors of urinary schistosomiasis and examined at small-scale the spatial heterogeneity in disease prevalence	Cross-sectional	Socio-demographics, parasitological and climatic factors	Lusaka	1. The risk factors were geographical location, altitude, normalized difference vegetation index, maximum temperature, age, sex of the child and IH snail abundance. 2. The mean prevalence rate was 9.6%. 3. Infection risk was highly correlated with IH host snail abundance and vegetation cover. 4. Location of the school (plateau or valley) had influence on the prevalence and intensity of infection.
Silwambe & Baboo (2009) [32]	The study determined the levels of knowledge and risk factors linked to the spread of the parasite among school children.	Cross-sectional	Socio-demographics and parasitological factors	Kaoma	1. The study observed that only 30% of the respondents had knowledge on the existence of the parasite. 2. The use of contaminated water for recreational purposes was found to be the main risk factor. 3. About 73% of the sampled respondents tested positive for *S. mansoni* and 21% were co-infected with both *S. mansoni* and hookworm.
Mutengo et al. (2010) [45]	The study determined and documented the presence of genital schistosomiasis from biopsy specimens	Cross-sectional	Socio-demographics, and histopathological factors	Samples collected from different parts of the country	1. The prevalence of female genital tract schistosomiasis was (84.2%). 2. Fifty three percent of females in the age group of 30–42 years had genital schistosomiasis. This was flowed by the age group of 17–29 years age group (25%). 3. Malignancy was clinically suspected in 74% of the genital schistosomiasis patients.
Agnew-Blais et al. (2010) [38]	The study determined the (i) prevalence of *S. haematobium* infection in a peri-urban school-aged population using the urinalysis (ii) the risk factors associated with infection in the study population (iii) the success of infection detection using in-school screening and examination processes	Observational/cross sectional	Socio-demographics and parasitological factors	Lusaka	1. Infection rate of 20.72% was observed in children between ages of 5 and 17 years. 2. Infection rates were higher for males (28.40%) than females (13.95%). 3. Detection of infection in the laboratory had a sensitivity of 24.70% and a specificity of 98.17% following suspicions.
Strahan et al. (2012) [34]	The study evaluated the prevalence of *Schistosoma mansoni*-related liver disease among school-age children living along the Zambezi River	Observational/cross sectional	Radiology and parasitological factors	Zambezi	1. Six students (1.5%) tested positive for *S. mansoni* eggs in their stool specimen. 2. 284 (37.2%) children were observed to be in danger of peri-portal fibrosis on ultra-sound. 3. Six (1.5%) were positive for *S. mansoni* eggs. 4. Four children were observed to be at risk of advanced fibrosis.
Payne et al. (2013) [33]	The study aimed at: (i)Assessing the burden of hepatosplenic pathology in Kaoma (ii) assess the prevalence of *Schistosoma mansoni* infections	Cross-sectional	Socio-demographics, clinical and parasitological factors	Kaoma	1. Ninety-seven (88%) respondents tested positive for *Schistosoma* antibodies. 2. Seventy-nine percent (*n* = 110) of the respondents tested positive for *schistosoma* antibodies in the blood Luampa while in Luena, 95% of the respondents were positive. 3. Forty-six (46) of the respondents reported blood in stool. 4. After clinical examinations, 27% of the respondents showed hepatomegaly, 17% splenomegaly, and 72% pallor.
Mutengo et al. (2014) [44]	The study aimed at determining the prevalence of *S. mansoni* infection and associated morbidity in four rural communities of western Zambia	Cross-sectional	Socio-demographics and parasitological factors	Kaoma	1. The burden of the disease in the study areas was high. 2. The prevalence of *S. mansoni* infection and geometric mean egg count (GMEC) were 42.4% and 86.6 eggs per gram of faeces, respectively. 3. Prevalence was highest in the age group of 15–19 years old. 4. Prevalence of fibrosis due to infection was high among female than male respondents.
Shawa et al. (2014) [35]	The study aimed at documenting the occurrence and prevalence on schistosomiasis and soil transmitted helminths (STHs) in some parts of Zambia	Cross-sectional	Socio-demographics and parasitological factors	Luangwa Kalabo Serenje	1. The prevalence of *S. haematobium* was generally low. Highest prevalence of 3.0% was recorded in Serenje. 2. In Kalabo, the prevalence of *S. mansoni* was 37.5%. 3. The prevalence of hookworms ranged from12.1 to 35.0% at all three sites. 4. Some of the STHs observed were *Ascaris lumbricoides, Hymenolepis nana* and *Enterobius vermicularis.*
Monde et al. (2016) [40]	The study examined the influence of environmental and socio-economic factors on the population dynamics of the intermediate host snails	Multi-level	Socio-economic and environmental factors	Sinazongwe Siavonga Solwezi Mufumbwe Zambezi	1. Gender significantly influences livelihood strategies. 2. Environmental parameters measured significantly influenced snail species composition, abundance and distribution. 3. Fifty-two (52%) and eighty-seven (87%) of the respondents in region I and III perceived that most schistosomiasis cases occurred during the hot season.
Halwindi et al. (2016) [31]	The study sought to determine the potential contribution of adult populations to the maintenance of schistosome and soil-transmitted helminth transmission	Cross-sectional	Socio-demographics and parasitological factors	Mazabuka, Siavonga	1. The prevalence of schistosomiasis among adults in Siavonga was 13.9%. 2. In the same area, the prevalence of *Ascaris lumbricoides* and hookworm was 12.1%. 3. No case of *S. mansoni* was observed in Mazabuka while the prevalence of S*. haematobium* was 5.3%. 4. The prevalence of *A. lumbricoides* and hookworm in the same area was 7.4%.
Simoonga & Kazembe (2017) [36]	The study quantified the risk factors associated with the intensity of urinary schistosomiasis infection among school in order to understand local transmission	Cross-sectional	Parasitological and climatic factors	Luangwa and Kafue	1. The risk of schistosomiasis infection was strongly influenced by age, altitude at which the child lived and sex. 2. Weak associations were observed with the normalized difference vegetation index, maximum temperature and snail abundance. 3. Infection intensity was reduced in the age group of 5 and 9 years. 4. The risks of infection were higher for children living in plateau areas than those living in valley areas.

Two studies were malacology-based and explored the distribution of schistosomiasis in relation to snail availability. Six studies explored the prevalence of schistosomiasis by focusing on evaluating risk factors and linking them to socio-economic/demographic factors. These studies further collected parasitological information to determine disease prevalence. Three studies were clinical-based: one study collected biopsy specimen for analysis and another was a radiologically focused study. The third study focused on assessing the burden of hepatosplenic pathology and prevalence of *S. mansoni* infections. Other remaining studies evaluated infection risks based on the presence of *Schistosoma* antibodies in humans. Only two studies applied geospatial technology in understanding the epidemiology of the disease.

**Fig. 2:**
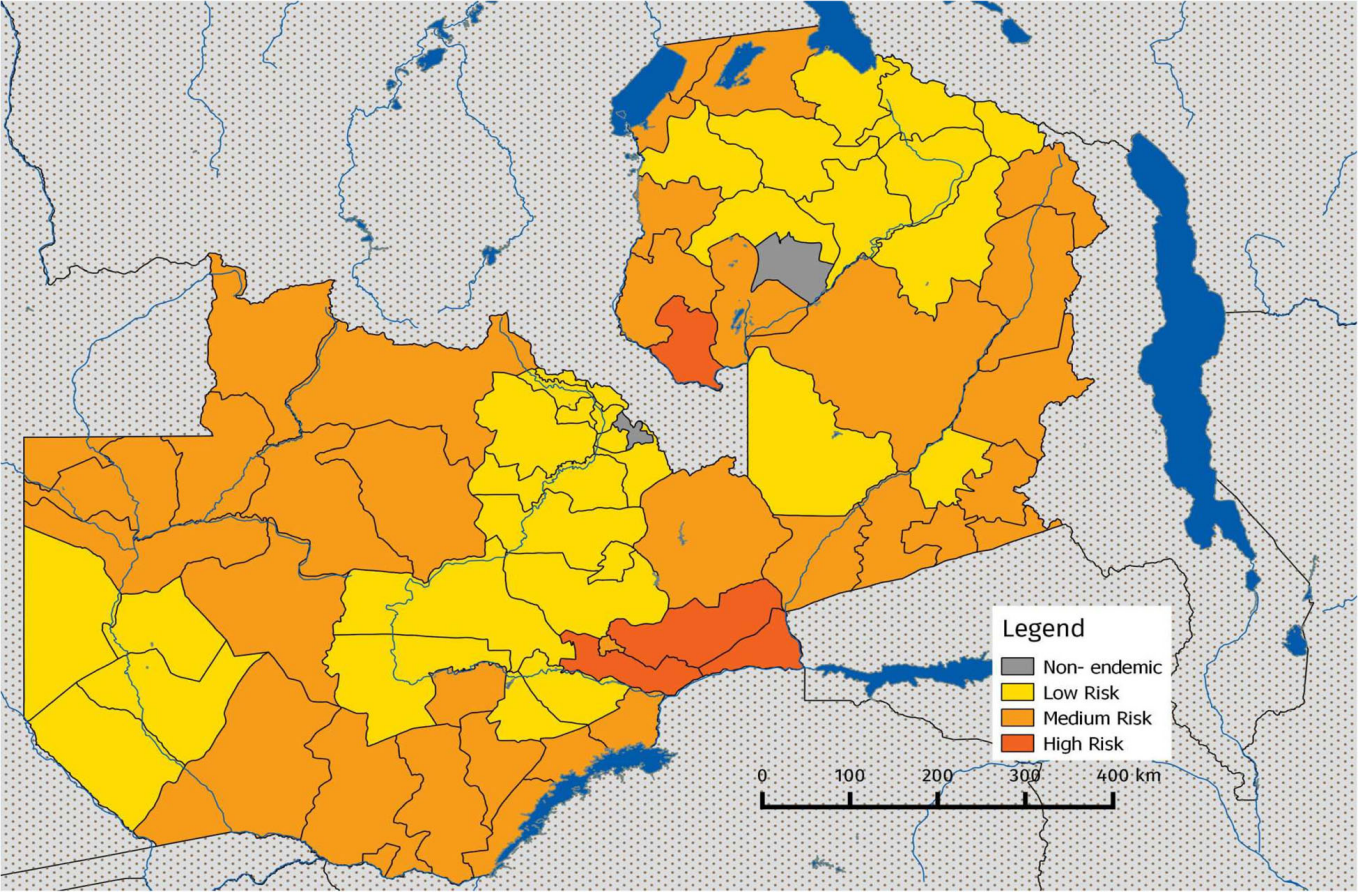
Map of Zambia showing the prevelance of schistosomiasis. Risk levels: low risk (≥1% and < 10%), medium risk (≥10% and < 50%) and high risk (≥50%). Source: Schistosomiasis Control Initiative [11]

The selected studies suggest that schistosomiasis is prevalent and endemic in the country (see Fig. 2), and there is need to establish its prevalence in various parts of the country. According to Mubila and Rollinson [30], the prevalence of *S. haematobium* infection around Lake Kariba was 76%. Halwindi et al. [31] reported that the prevalence of the disease in Siavonga was 13.9%, with *S. haematobium* more prevalent than *S. mansoni*. Studies done in the western province reported more cases of *S. mansoni* than *S. haematobium*, with *S. mansoni* prevalent in 73% of the sampled individuals [32]. This is further reflected in the clinical-based study conducted by Payne et al. [33] which reported that 97% of the respondents tested positive for *Schistosoma* antibodies in their blood samples. The presence of schistosomiasis has also been reported in Zambezi, north-western province, where 37.2% of the children sampled were also in danger of peri-portal fibrosis due to *S. mansoni* infection [34]. Schistosomiasis has also been reported in Serenje (central province) [35] and in Lusaka [36–38] where prevalence rates of about 90.1% were observed in some communities [39] (see Table 1 and Fig. 2).

Various approaches have been used to study the epidemiology of schistosomiasis in Zambia. Monde et al. [40] explored the influence of various environmental factors on the abundance of IH snails, while Simoonga et al. [37] established a link between climatic variables and disease prevalence using a spatial-epidemiology approach. The study concluded that altitude, normalised difference vegetation index (NDVI), maximum temperature, age, sex of the child and IH snail abundance were the main factors contributing to disease prevalence. A statistical modelling approach using a hierarchical ordinal regression model was used by Simoonga and Kazembe [36] to further understand the influence of age, sex and altitude at which the child lived, NDVI, maximum temperature and snail abundance on infection risk. (see Table 1).

Although all studied have reported cases of schistosomiasis in both pre-urban and rural parts of Zambia, only Monde et al. [40], Chimbari et al. [41] and Mubila and Rollinson [30] have reported on the presence of IH snails.

## Discussion

### Biogeography of schistosomiasis in Zambia

Despite the implementation of disease control programmes [39] and the prospects of scaling them up [10], the prevalence of schistosomiasis may be higher than previously reported by Chitsulo et al. [13] and Zambia Bilharzia Control Programme [42]. Furthermore, schistosomiasis is prevalent and endemic across all 10 provinces [11] with *S. haematobium* endemic in 69 districts and *S. mansoni* in 49 districts [39]. The increase in the prevalence may be attributed to climate change [5], changes in livelihood strategies [40], religious and cultural practices which may influence human-water contact behaviours (e.g. baptism by immersion) and health-seeking behaviours [8, 38] and potentially perpetuate the maintenance of the parasite within the population [31].

The current study also indicates that the burden of morbidities associated with schistosomiasis in Zambia has not been fully explored. According to King and Dangerfield-Cha [43], schistosomiasis may present long-term health threats to the populations. Cases of peri-portal fibrosis among rural children in north-western province [34], peripheral fibrosis and main portal branch fibrosis in western province due to *S. mansoni* infection have been reported [44]. Additionally, Mutengo et al. [45] observed the presence of squamous cell carcinoma in cervical biopsies and genital malignancy among *S. haematobium*-infected individuals, which increases the risks of cervical cancer. Furthermore, infection with *S. haematobium* among women may lead to female genital schistosomiasis (FGS) [46], increase the risk of impaired reproductive organs [47] and HIV infection [48, 49]. The observed high prevalence of schistosomiasis in Zambia may indicate increased risk of both FGS and HIV infection especially among the women. This outcome suggests a need for guidelines and policy change in chemotherapy by also targeting the adult population group besides the school-age based population which for long has been the focus [50].

### Scope of schistosomiasis studies conducted in Zambia since 2000

Many studies have evaluated the prevalence of schistosomiasis and its associated risk factors. Monde et al. [40] showed the relationship between various environmental factors and snail abundance. Other studies have also suggested that the transmission of schistosomiasis can also be influenced by IH snail-and parasite-related factors [51], temperature [7, 52, 53] and rainfall [54].

This review indicates that schistosomisis occurs within the socio-ecological system, therefore institutional factors are vital both in the spread and elimination of the diseases. Institutional factors such as policies, health infrastructure and developmental patterns may have long-and short-term effects on the economies of local communities and risk of infection [55]. These factors may influence the utilisation of health resources, and the development and deployment of effective control programmes. The current review suggests that there is a need to look at the epidemiology of schistosomiasis and contextualise aspects of institutional and ecological factors to suit the current disease control and elimination programmes.

### Environmental factors and disease distribution

The effect of seasonal changes, ecology and the environment on schistosomiasis transmission in Zambia remains poorly understood. According to Utzinger et al. [56], ecological factors greatly influence the presence and abundance of IH snails and the development of the cercariae within them. Shiff et al. [57] and Manyangadze et al. [4] highlighted the seasonal population dynamics of IH snails. These studies indicated that the snail population reduces during the rain-season due to running water washing away the snails [58], while high water temperature and drying up of temporal water ponds induces mortality during the dry, hot seasons [29, 58], which also decreases the size of the snail population. Understanding these dynamics may aid efficient application of molluscicides and other snail control measures [57, 59].

In the study conducted by Simoonga et al. [37], the results showed that NDVI values between 128 and 160, and maximum temperatures of 20 °C to 21 °C increased the infection risk and enhanced the development of the snail-parasite system. This was corroborated by the findings of Manyangadze et al. [4] and Mukaratirwa et al. [60]. Except for studies conducted by Simoonga et al. [37] and Monde et al. [40], there is limited data on snail ecology, and environmental and climatic factors related to schistosomiasis transmission in Zambia. To effectively control schistosomiasis, MDA programmes should be accompanied by knowledge on the distribution and behaviour of IH snails [24, 61, 62]. This study therefore suggests the need of adopting recommendations by the Special Programme for Research and Training in Tropical Diseases to revive vector studies including snails [63, 64] within the country’s schistosomiasis programmes.

### Using ecological models

The use of geographic information system (GIS) has been found to be essential in evaluating the suitability of specific areas for the development of the snail-parasite system, and monitoring of snail control programmes [24]. This is essential at the local level in Zambia where up-to-date parasitological data may not be readily available or difficult to access. Furthermore, the incorporation of GIS in predictive models has enhanced development of snail habitat suitability and distribution maps. These maps can be combined with suitable temperature regimes to define possible areas for the development of the snail-trematode system [24]. Such initiatives may increase the chances of successful implementation of health interventions in the context of limited resources.

Modelling approaches such as those explored by Simoonga et al. [37] and Simoonga and Kazembe [36] used robust statistical methods to explore possible uncertainties that may be associated with schistosomiasis intensity in Zambia. This review encourages the use of such models that account for the distribution of IH snails based on habitat suitability and intra-molluscan parasite development. Furthermore, modelling approaches used by Zhou et al. [2] and McCreesh et al. [5] to predict future changes in the geographical spread of IH snails are encouraged for effectively planning snail and disease control measures.

### Policy implications

Schistosomiasis has been listed by WHO as a NTD, and there is need to eliminate it. This review has shown that the disease is endemic in Zambia and the need for treatment remains high. The new National Health Strategic Plan (2017–2021) acknowledges the impact of schistosomiasis and other NTDs, thus necessitating the need for national implementation of MDA.

In line with the recommendations by WHO, this review suggests the need to precisely map the distribution of the disease and IH snail distribution to support an effective MDA programme. The emphasis on snail populations is a recognition that snail populations may indicate potential transmission areas. Interventions targeted at snails in certain localities are possible. For instance, effective management of water for snail control was done in Zimbabwe (Mushandike Irrigation Scheme) [65]. The observed high prevalence of schistosomiasis among rural communities may indicate insufficient sanitary facilities and the use of contaminated water for domestic and recreational activities [31, 40, 25, 44].

### Insights for public health professionals and possible directions for future control strategies

1. There is a need to tailor studies on understanding the probable impact of climate change on schistosomiasis risks. Human-driven anthropogenic activities and environmental change may alter the disease patterns and geographical range of IH snails [1, 2, 22]. Our review recommends the integration of socio-demographics, as well as environmental and IH snail-related factors to provide a better understanding of the possible impact of climate change on disease risks.
2. There is a need to develop predictive models that can be used to delineate present and potential future distribution and snail suitability areas to inform control measures. Due to a paucity of parasitological data, which makes determination of the actual disease prevalence difficult, predictive models that make use of both field-based and laboratory studies, as well as snail habitat suitability maps may be useful for indicating potential areas for disease risks. This will improve the design, implementation and monitoring of disease control measures.
3. Using the prevalence distribution map that was developed by the Schistosomiasis Control Initiative, there is a need to strengthen community engagement strategies through health education. Local-level institutions may also be strengthened to reduce the perpetuation of the parasite within the adult population [31, 50].

## Conclusions

It is evident that schistosomiasis is prevalent in Zambia, and there is a need for researchers and disease control managers in the country, through inter-sectoral collaboration, to devise new control strategies and periodic disease surveillance programmes. This will increase the success of MDA programmes, which will be informed by disease distribution patterns. Furthermore, this review suggests the need to develop a specific action plan against schistosomiasis and devise applicable control measures for both children and adults to reduce the perpetuation of the parasite in the communities. Schistosomiasis occurs in the socio-ecological system, and therefore understating the epidemiology of the disease requires going beyond social determinants and including climatic variability and epidemiological niche modelling.

## Additional file

Additional file 1: Multilingual abstract in the five official working languages of the United Nations. (PDF 762 kb)

## Abbreviations

FGS: Female genital schistosomiasis
GIS: Geographic information system
IH: Intermediate host
MDA: Mass drug administration
NDVI: Normalised difference vegetation index
NTD: Neglected tropical disease
WHO: World Health Organization
ZBCP: Zambia Bilharzia Control Programme
