# Supplementary material for: Schistosomiasis in Zambia: a systematic review of past and present experiences
Source: Infect Dis Poverty. 2018 Apr 30;7:41. doi: 10.1186/s40249-018-0424-5 (PMC5925830; doi:10.1186/s40249-018-0424-5)
Supplement: Supplementary file 1 — Multilingual abstract in the five official working languages of the United Nations. (PDF 762 kb) [file 40249_2018_424_MOESM1_ESM.pdf]

Translation of the abstract into the five official working languages of the United Nations

داء البلهارسيا في زامبيا: مراجعة منهجية للخبرات الماضية والحالية

تشيستر كاليندا، موسى جي، تشيمباري، سامسون موكاراتيروا

الملخص

الخلفية: قد يزيد معدل التغير السريع في العوامل البيئية والاجتماعية-الاقتصادية من حدوث وانتشار خطر الإصابة بعدوى البلهارسيا في زامبيا. ومع ذلك، فإن المعلومات المتاحة لا توفر فهماً شاملاً للجغرافيا البيولوجية وتوزيع المرض، وعلم البيئة وديناميكيات التعداد للعوائل الوسيطة المضيفة. اتبعت الدراسة الحالية نهجًا نظريًا للمعلومات من أجل فهم الجغرافيا الحيوية ومدى انتشار داء البلهارسيا وتحديد الفجوات المعرفية التي قد تكون مفيدة لتحسين سياسة المراقبة والقضاء على العوائل الوسيطة في زامبيا.

الطرق: لتلخيص المعارف المتوفرة والبناء على التجارب السابقة والحالية لوبائيات البلهارسيا من أجل مكافحة فعالة للمرض في زامبيا، تم إجراء بحث منهجي للمطبوعات للفترة 2000–2017 على PubMed وGoogle Scholar وEBSCOhost. باستخدام الكلمات الرئيسية: *"البلهارسيا"*، *'بيومفلاريا'*، *'بولاينس'*، *'البلهارسيا المنسونية'*، *'البلهارسيا الدموية'*، و *"زامبيا"*، بالاشتراك مع المصطلحات البوليانية "AND" و "OR"، تم الحصول على تقارير / أبحاث منشورة ومراجعتها بشكل مستقل لتضمينها.

النتائج: تم اختيار ثلاثة عشر بحثًا باللغة الإنجليزية والتي استوفت معايير الاشتمال للمراجعة النهائية. تشير الأبحاث إلى أن خطر العدوى قد ازداد على مر السنين، ويعزى ذلك إلى عوامل بيئية واجتماعية واقتصادية وديموغرافية. وعلاوة على ذلك، فإن داء البلهارسيا متوطن في أجزاء كثيرة من البلاد بسبب أن الإصابة بـ*البلهارسيا الدموية* أكثر انتشاراً من *البلهارسيا المنسونية*. وجدت هذه المراجعة أيضًا أن البلهارسيا الدموية ترتبط بالآفات التناسلية، مما يزيد من خطر الإصابة بأمراض أخرى مثل فيروس نقص المناعة المكتسبة وسرطان عنق الرحم.

الاستنتاج: بالنسبة لكل من *البلهارسيا الدموية والبلهارسيا المنسونية* على حد سواء، كانت العوامل البيئية والاجتماعية-الاقتصادية والديموغرافية مؤثرة في انتقال وانتشار المرض وتسليط الضوء على الحاجة إلى معرفة مفصلة عن النمذجة البيئية ورسم خرائط توزيع المرض والعوائل الوسيطة المضيفة من أجل تنفيذ فعال لاستراتيجيات التحكم.

Translated from English version into Arabic by Free Bird, proofread by Abdessalam AIT TOUIJAR, through

TRANSLATORS WITHOUT BORDERS

赞比亚血吸虫病：对过去和现在经验的系统综述

Chester Kalinda, Moses J. Chimbari, Samson Mukaratirwa

摘要

引言：环境和社会经济因素的迅速变化可能会提高赞比亚血吸虫病的发病率和流行率，增加感染风险。然而，现有资料不能全面反映中间宿主钉螺的生物地理学和疾病分布，以及生态学和种群动态。本研究采用信息论方法以了解赞比亚血吸虫病的生物地理学和流行情况，并确定了有助于改进钉螺监测和消除政策的知识空白。

方法：为了总结已有知识，借鉴过去和现在赞比亚有效控制血吸虫病的经验，本研究在 PubMed、Google Scholar 和 EBSCOhost 数据库对 2000-2017 年的文献进行系统检索。使用关键词"Schistosomiasis"、"*Biomphalaria*"、"*Bulinus*"、"*Schistosoma mansoni*"、"*Schistosoma haematobium*"和"Zambia"，结合布尔表达式"AND"和"OR'"，独立获取已发表的报告/论文并进行审查以供纳入。

结果：筛选出 13 篇符合纳入标准的英文文章进行最终评审。这些文章表明，多年来感染风险有所增加，归因于

环境、社会经济和人口因素。此外，血吸虫病在赞比亚许多地方流行，相较于曼氏血吸虫，埃及血吸虫感染更为普遍。本综述还发现，埃及血吸虫与生殖器病变有关，从而增加了罹患艾滋病和宫颈癌等其他疾病的风险。
结论：环境、社会经济和人口因素对埃及血吸虫病和曼氏血吸虫病的传播和流行都有影响。需应用生态建模知识绘制疾病和中间宿主钉螺的分布图，以便有效实施控制策略。

Translated from English version into Chinese by Pei Wang, edited by Pin Yang

CHINESE CENTER FOR DISEASE CONTROL AND PREVENTION
NATIONAL INSTITUTE OF PARASITIC DISEASES

La schistosomiase en Zambie : revue systématique des expériences passées et présentes

Chester Kalinda, Moses J. Chimbari, Samson Mukaratirwa

Résumé
Contexte: La rapidité de changement des facteurs écologiques et socioéconomiques peut accroître l’incidence, la prévalence et le risque de schistosomiase en Zambie. Toutefois, les informations disponibles ne permettent pas de comprendre complètement la biogéographie et la distribution de la maladie, ni l’écologie et la dynamique de population des gastéropodes qui en sont les hôtes intermédiaires. La présente étude a utilisé une approche par la théorie de l’information afin de comprendre la biogéographie et la prévalence de la schistosomiase. Elle a identifié des lacunes de connaissances qu’il serait utile de combler afin d’améliorer la surveillance et l’éradication des hôtes intermédiaires en Zambie.
Méthodes: Une recherche dans la littérature de 2000 à 2017 a été effectuée dans PubMed, Google Scholar et EBSCOhost afin de collecter les connaissances existantes et de profiter des retours d’expériences passées et actuelles sur l’épidémiologie de la schistosomiase dans le but de lutter efficacement contre la maladie en Zambie. Les articles et rapports publiés ont été recherchés à l’aide des mots-clés « Schistosomiasis », « *Biomphalaria* », « *Bulinus* », « *Schistosoma mansoni* », « *Schistosoma haematobium* » et « Zambia » et examinés indépendamment en vue de leur inclusion.
Résultats: Treize articles publiés en anglais satisfaisaient les critères d’inclusion et ont été sélectionnés pour la revue finale. Ces articles suggèrent que le risque d’infection a augmenté avec les années et attribuent cette augmentation à des facteurs écologiques, socioéconomiques et démographiques. En outre, la schistosomiase est endémique dans de nombreuses régions du pays, où l’infestation par *Schistosoma haematobium* est plus fréquente que par *S. mansoni*. Cette revue a également révélé que *S. haematobium* était associé à des lésions génitales et augmentait ainsi le risque de contracter d'autres maladies telles que le VIH et le VPH (cancer du col de l’utérus).
Conclusion: Pour *S. haematobium* comme pour *S. mansoni*, des facteurs écologiques, socioéconomiques et démographiques ont influé sur la transmission et la prévalence de la schistosomiase. Il apparaît nécessaire de disposer de connaissances détaillées en vue de la modélisation écologique et la cartographie de la distribution de la maladie et des gastéropodes qui en sont les hôtes intermédiaires, dans la perspective d’une mise en œuvre efficace des stratégies de lutte.

Translated from English version into French by Suzane Assenat, proofread by Rodolphe Blet, through

TRANSLATORS
WITHOUT BORDERS

Шистосомоз в Замбии: систематический обзор прошлого и настоящего опыта

Честер Калинда, Мозес Дж. Чимбари, Самсон Мукаратирва

Аннотация

Краткое описание. Быстрые темпы изменения экологических и социально-экономических факторов способны повысить заболеваемость, распространённость и риск заражения шистосомозом в Замбии. При этом доступная информация не обеспечивает всестороннего понимания биогеографии и распространения заболевания, а также экологии и динамики популяции улиток, являющихся промежуточным носителем. В настоящей работе задействован информационно-теоретический подход к пониманию биогеографии и распространения шистосомоза, и выявлены пробелы в знаниях, необходимых для улучшения политики по надзору и устранению промежуточного носителя - улитки на территории Замбии.

Методы. Для обобщения существующих знаний и учёта прошлого и текущего опыта эпидемиологии шистосомоза с целью эффективной борьбы с заболеванием в Замбии был осуществлён систематический поиск литературы за период с 2000 по 2017 год в PubMed, Google Scholar и EBSCOhost. В результате поиска по ключевым словам: «Schistosomiasis» (шистосомоз) ,*«Biomphalaria» (биомфалярия*),*«Bulinus» (булинус)*, *«Schistosoma mansoni» (шистосома Мансона)*, *«Schistosoma haematobium» (мочеполовая шистосома*) и «Zambia» (Замбия) в сочетании с логическими союзами «AND» (И) и «OR» (ИЛИ), были получены опубликованные отчёты и статьи, по которым был произведен независимый обзор на предмет их включения.

Результаты. Для заключительного обзора были отобраны тринадцать научных статей на английском языке, которые соответствовали критериям включения. По данным научных статей с годами наблюдается повышение риска заражения, что обусловлено экологическими, социо-экономическими и демографическими факторами. Более того, в ряде районов страны шистосомоз эндемичен, при этом заражение *мочевополовым шистосомозом* преобладает над заражением, обусловленным *шистосомой Мансона*. Настоящий обзор выявил связь *мочеполового шистосомоза* с поражением половых органов, в результате чего повышается вероятность заражения другими заболеваниями, такими как ВИЧ и рак шейки матки.

Заключение. Как в случае с *мочеполовым шистосомозом,* так и с *шистосомой Мансони,* экологические, социо-экономические и демографические факторы оказали большое влияние на передачу и распространённость заболевания, что подчёркивает необходимость более глубоких знаний в области экологического моделирования и картирования распространения заболевания и его промежуточного носителя - улитки с целью эффективного применения стратегий по борьбе с заболеванием.

Translated from English version into Russian by Liudmila Tomanek, proofread by Natalia Potashnik, through

TRANSLATORS
WITHOUT BORDERS

La Esquistosomiasis en Zambia: Un análisis sistemático de experiencias pasadas y presentes.

Chester Kalinda, Moses J. Chimbari, Samson Mukaratirwa

Resumen

Antecedentes: La rápida tasa de cambio en los factores ambientales y socio-económicos puede aumentar la incidencia, la prevalencia y el riesgo de infecciones por esquistosomiasis en Zambia. Sin embargo, la información disponible no proporciona una comprensión exhaustiva de la biogeografía y la distribución de la enfermedad, la ecología y la dinámica de la población de los caracoles huéspedes intermedios. En el estudio actual se utilizó un enfoque teórico-informativo para entender la biogeografía y la esquistosomiasis prevalente, y se identificaron carencias en el conocimiento que serían útiles para mejorar las políticas de vigilancia y erradicación de los caracoles huéspedes intermedios en Zambia.

Métodos: Para resumir el conocimiento existente y aprovechar las experiencias pasadas y presentes de la epidemiología de la esquistosomiasis para un control eficaz de la enfermedad en Zambia, se realizó una búsqueda sistemática de literatura del período 2000-2017 en PubMed, Google Scholar y EBSCOhost. Usando las palabras clave: 'Schistosomiasis', '*Biomphalaria*', '*Bulinus*', '*Schistosoma mansoni*', '*Schistosoma haematobium*', y 'Zambia', en combinación con los términos de Booleans 'AND' and 'OR', informes/documentos publicados fueron obtenidos y revisados de forma independiente para su inclusión.

Resultados: Se seleccionaron trece artículos publicados en inglés que cumplían con los criterios de inclusión para el análisis final. Los artículos sugieren que el riesgo de infección ha aumentado con los años y esto se atribuye a factores ambientales, socio-económicos y demográficos. Además, la esquistosomiasis es endémica en muchas partes del país y las infecciones causadas por*Schistosoma haematobium* son más comunes que aquellas causadas por *S. mansoni* . Este análisis también encontró que *S. haematobium* está relacionado con lesiones genitales, incrementando así el riesgo de contraer otras enfermedades como VIH y cáncer cervical.

Conclusión: Para ambos *S. haematobium* y *S. mansoni*, factores ambientales, socio-económicos y demográficos influyeron en la transimisión y prevalencia de la enfermedad y se recalca la necesidad de un conocimiento más detallado sobre la modelización ecológica y la cartografía de la distribución de la enfermedad y los caracoles huéspedes intermedios para una aplicación efectiva de estrategias de control.

Translated from English version into Spanish by ximeniuts, proofread by Concepción Martín, through

TRANSLATORS
WITHOUT BORDERS
